# Usefulness of a Bispectral index oriented sedative method without neuromuscular blocker for therapeutic hypothermia after cardiac arrest

**DOI:** 10.1186/cc10893

**Published:** 2012-03-20

**Authors:** S Shiraishi, Y Ohta, T Tagami, Y Ono, T Masuno, H Yokota

**Affiliations:** 1 Nippon Medical School, Tokyo, Japan

## Introduction

During therapeutic hypothermia (TH) after cardiac arrest (CA), neuromuscular blockers are often used to prevent or treat thermogenic shivering [1]. But the following risks due to neuromuscular paralysis are encountered: prolonged muscle weakness, hypostatic pneumonia and venous thromboembolism. So we evaluated the usefulness of Bispectral index (BIS) oriented sedation without neuromuscular blocker in six cases of post CA patients receiving TH.

## Methods

Six consecutive patients admitted after CA and treated with TH by the same attending physicians' group were included. BIS monitoring was applied immediately after the admission to ER. After initial resuscitation and radiological examination, including coronary angiography and angioplasty, patients were admitted to the ICU and cooled down to a target body temperature of 34°C using a surface cooling system with an external pad. Target body temperature was maintained for 48 hours and rewarmed to 36°C over another 48 hours. As induction of patients' sedation, we injected 5 mg midazolam and 0.2 μg fentanyl intravenously just as we recognized patients' movement or immediately before induction of TH. For maintenance of sedation, midazolam at dose 0.1 mg/kg/hour, dexmedetomidine at dose 0.4 μg/kg/hour and fentanyl at doses 0.8 μg/kg/hour were administrated continuously. The midazolam and the dexmedetomidine infusion were adjusted to a target BIS value of 40 or less. BIS monitoring was ceased after completion of both rewarming and discontinuation of sedative drugs.

## Results

In all six patients, TH was completed without severe complication, especially shivering movement and serious hypostatic pneumonia. Three patients presenting unstable BIS values lower than 10 during TH showed poor neurological outcome, while the other three patients presenting stable BIS values about 40 showed favorable neurological outcome. Myoclonic movement or convulsion, regarded as signs of bad outcome, was observed in two poor neurological outcome patients. Cough reflex was observed in two favorable neurological outcome patients throughout their TH.

## Conclusion

BIS oriented sedation without neuromuscular blocker is feasible in maintaining TH for survivors from CA. By keeping muscular function, both noxious and beneficial movements are preserved and these help us to predict neurological outcome and prevent patients from hypostatic disorders.
